# Body Condition Score Is Not Correlated to Gastric Ulcers in Non-Athlete Horses

**DOI:** 10.3390/ani12192637

**Published:** 2022-09-30

**Authors:** Sara Busechian, Luca Turini, Micaela Sgorbini, Francesca Bonelli, Lorenzo Pisello, Camillo Pieramati, Simona Orvieto, Fabrizio Rueca

**Affiliations:** 1 Department of Veterinary Medicine, University of Perugia, Via San Costanzo 4, 06126 Perugia, Italy; 2 Department of Agricultural, Food and Agro-Environmental Sciences, Via del Borghetto 80, 56124 Pisa, Italy; 3 Department of Veterinary Sciences, University of Pisa, Viale delle Piagge 2, 56124 Pisa, Italy; 4 Independent Researcher, 06126 Perugia, Italy

**Keywords:** body condition score, equine gastric ulcer syndrome, equine squamous gastric disease, equine glandular gastric disease

## Abstract

**Simple Summary:**

Equine gastric ulcer syndrome (EGUS) is a worldwide disease, quite common in various categories of horses. Numerous clinical signs have been reported, including inappetence, poor performance, girthiness, and recurrent colic. In addition, weight loss and a low body condition score (BCS) are considered to be associated with EGUS. However, few studies have examined the relationship between BCS and gastric ulcers, and the results were contradictory. Gastroscopies were performed on 203 horses to detect the presence of gastric ulcers. At the same time, a board-certified veterinarian, blinded to the results of the gastroscopies, assessed the BCS of the horse. In our population, BCS was not correlated with the presence or severity of gastric ulcers. Gastric ulceration, therefore, cannot be excluded based only on the presence of a normal or high BCS.

**Abstract:**

Equine Gastric Ulcer Syndrome (EGUS) is a worldwide disease of the stomach that can be found in different categories of horses. Different clinical signs may be present, but a large number of horses are asymptomatic. The aim of this study was to identify a possible correlation between body condition score (BCS) and EGUS in a population of horses. A total of 203 non-athlete horses were submitted for gastroscopies, and the presence and severity of lesions of the glandular and squamous mucosa were recorded. A board-certified veterinarian blinded to the gastroscopy results assessed the BCS of the horse. In the study population, no correlation was found between BCS and the presence of gastric lesions in either the glandular or the squamous mucosa. The disease of the squamous or glandular mucosa cannot be excluded based only on the presence of normal or increased BCS in non-athlete horses.

## 1. Introduction

Equine gastric ulcer syndrome (EGUS) is a worldwide disease in equids, described in different populations with different prevalence [1,2,3,4,5,6,7,8,9,10,11,12,13]. The ECEIM Consensus Statement divided the disease into two different entities based on the involvement of either the squamous (equine squamous gastric disease, ESGD) or the glandular mucosa (equine glandular gastric disease, EGGD) [1,2,6,7,13]. Both disease entities have been associated with various clinical signs, ranging from exercise intolerance and poor performance to weight loss, inappetence, and recurrent colic. Many horses, however, do not show symptoms of disease [1,2,5,6,7,14,15]. The body condition score (BCS) relies on the evaluation of specific parts of the body to determine the amount of fat present. Various grading systems have been used throughout the years: low levels are indicative of emaciation, while higher is correlated with overconditioning and obesity [16,17,18,19]. A relationship between BCS and measured carcass fat depots was identified in a recent study, indicating that BCS may be a reliable method for assessing horses’ fat deposits [20]. Moreover, BCS is easy to evaluate, especially on site, where more sophisticated methods, such as ultrasonography, are not always available [16]. The BCS may be used as a screening tool to detect weight loss due to decreased energy intake or high energy demand. A low BCS can be caused by various conditions and diseases, such as lack of access to appropriate food or difficulty in ingesting nutrients in feed, but also abnormal digestion or metabolism or reduced delivery of nutrients to peripheral tissues. On the other hand, horses can have increased energy or protein demands that cannot be met by normal maintenance rations. This can happen in elite sports and racehorses under heavy training [19]. EGUS has been associated with different symptoms, including, among others, poor appetite or “picky eating”, poor BCS or weight loss, chronic diarrhea, and acute or recurrent colic [1,2,6,7,13]. Low BCS in horses with gastric ulcers could be related to the presence of recurrent colic, that can be seen especially after eating: this can lead to “picky eating”, decreased nutrient uptake and weight loss [1,2]. A relationship between BCS and the presence of gastric ulcers in racehorses in training, Standardbred in particular, has been found [5]. This category of horses, though, is subjected to high levels of exercise and increase stress, related to their management, the level of exercise and stress [12]. No information is available about the relationship between BCS and gastric ulcers in either the glandular or the squamous mucosa in pleasure or retired horses. The aim of this study was to investigate the relationship between BCS and ESGD or EGGD in a cohort of non-athlete horses.

## 2. Materials and Methods

In this study, a cohort of horses meeting at least one of the following inclusion criteria were enrolled: (1) horses showing clinical signs compatible with gastric lesions (recurrent colic, poor appetite or picky eating or weight loss, behavioral signs); (2) horses subjected to risk factors commonly associated with EGUS (e.g., changes in management or diet, pharmacological treatments, etc.) [1,2,6,7,12,21,22]. All horses were presented for evaluation at the request of the owner or the referring veterinarian, and gastroscopy was performed as part of the diagnostic workup. Exclusion criteria: horses used for racing or in heavy training (i.e., endurance horses competing at the international level, show-jumpers during the peak of international season).

For all horse, a complete physical, dental, and fecal egg count examination were performed, and animals presenting any abnormality were treated as appropriate (i.e., deworming treatment as necessary, dental care, etc.) before performing gastroscopy. All animals were vaccinated according to international guidelines [23].

Gastroscopy was performed according to the literature [1,24], using a 300 cm long endoscope (60130PKS, Karl Storz Endoscope, Tuttlingen, Germany) and a portable processor (Tele Vet X Led, Karl Storz Endoscope). The examination was recorded on digital media for evaluation and storage. Food was withheld for at least 18 h and water for at least 4 before the examination. After sedating the animals with xylazine (0.25–1.1 mg/kg), the endoscope was introduced in the ventral nasal meatus and the pharynx and advanced into the esophagus to reach the stomach. All regions of the stomach were visualized after rinsing the mucosa with water as needed. ESGD and EGGD were assessed as previously reported [1,2,12]. Briefly, lesions in the squamous mucosa were graded from 0 to 4/4, based on the macroscopic appearance, with grade 0 given when no lesions were present and grade 4 when areas of deep ulceration were visible; ESGD was considered positive when at least grade 2/4 lesions were recorded. For the glandular mucosa, because of the lack of a validated grading system, the appearance of the mucosa was described, and horses were considered positive for EGGD when any irregularity (e.g., hyperemia of the mucosa, raised and fibrinosuppurative or flat and hemorrhagic lesions) was detected [1,2,12].

BCS was assessed and scored according to the literature [17] by a board-certified Equine Internal Medicine Specialist (S.B.) blinded to the gastroscopy results.

Statistical analysis was performed to determine the correlation between BCS and presence (Wilcoxon test) and grade (Spearman test) of ESGD and BCS and EGGD (Wilcoxon test). A chi-square test has been used to evaluate the relationship between BCS (classified in ≤2, 3, and ≥4) and the presence of ESGD. The analysis was carried out using R software (The R Foundation, Austria). Differences between means were declared significant at *p* ≤ 0.05.

## 3. Results

A total of 203 horses met the inclusion criteria and were included in this study. Overall, 8/203 (3.9%) horses included were Baroque (e.g., Lipizzaners, Friesian, etc.), 129/203 (63.6%), fullblood (e.g., thoroughbred, Arabians, quarter horses, etc.), and 66/203 (32.5%) warmblood (e.g., saddlebred) [12]. Of the 203 horses, 127/203 (62.6%) were females, 54/203 (26.6%) geldings, and 22/203 (10.8%) males. The horses were between 2 and 26 years old, with a median age of 9 years old. Most of the horses did not perform any exercise and were either retired, resting for several months before returning to active work, or were used for breeding purposes (females or stallions), while 77/203 (38%) were ridden in an arena or for short trekking once a week or less. All animals were kept in paddock or pasture, alone or in small groups, for at least a few hours every day. All horses were fed 8–10 kg of grass hay two to three times a day, with a small amount (1–2 kg/day) of sweet feed (mixed of grains, corn, bran, molasses, minerals, and vitamins) administered once or twice a day.

ESGD was present in 137/203 horses (67.5%): lesions were mostly found on the lesser curvature, near the margo plicatus, but the less severe ulcers were visible also along the greater curvature. On the glandular mucosa, lesions were present in 52/203 horses (25.1%), limited to areas of hyperemia, especially along the greater curvature. The distribution of the horses according to the relationship between BCS and ESGD and EGGD can be found in Table 1 and Table 2, respectively.

No correlation has been found between BCS and presence of ESGD (Wilcoxon rank sum test, *p* = 0.61), grade of ESGD (Spearman correlation, *p* = 0.56) or EGGD (Wilcoxon rank sum test, *p* = 0.74). No relationship has been found between the different classifications of BCS (≤2, 3, and ≥4) and the presence/absence of ESGD (*p* = 0.26).

## 4. Discussion

EGUS is a disease that occurs worldwide and affects different horse populations with different prevalence and different symptoms, among which, according to the literature, poor BCS and weight loss, poor appetite, diarrhea, or colic [1,2,6,7,13]. Risk factors for gastric ulcers have been widely studied in various horse populations, and despite our animals not being in active heavy training, a high proportion of them presented ESGD. This is in line with current literature findings [1,2,3,6,7,12,15] and highlights the need for further studies on the presence of EGUS in pleasure, retired or non-athlete horses. The aim of this study was to verify a possible relationship between BCS and ESGD or EGGD presence and ESGD score in a cohort of non-athlete horses performing minimal or no exercise. Overall, no relationship was found between BCS and the presence of gastric lesions in either the squamous (ESGD) or the glandular mucosa (EGGD) or between BCS and ESGD scores. The lack of a relationship was in contrast with previously published data [1,2,5,15]. In these previous studies performed on racehorses in active training, lower BCS was associated with the presence of gastric ulceration, and inappetence is considered a sign of EGUS [1,2,5,7,25,26]. In a study by Niedźwiedź et al. [15], the prevalence of gastric ulcers in pleasure horses with clinical signs (weight loss and poor BCS included) was higher compared with animals without symptoms. In this paper, BCS was not quantified by either the owner or the Veterinarian. Other authors, however, point out that gastric ulcers can be present without clinical signs [1,2,6,7,21]. This difference in our population could be related to the animals evaluated; in particular, most of the owners did not report clinical signs and asked for a complete evaluation of the gastrointestinal tract due to the presence of risk factors for EGUS [1,2,12,21,22]. In our population of non-athlete horses, though, poor performance or girthiness can be difficult to evaluate, especially in animals managed at pasture. Living on pasture and with other horses could mask the presence of inappetence. Not all owners are able to correctly evaluate the BCS of their horses, making it difficult for them to detect the presence of weight loss at an early stage [27,28,29,30]. Nevertheless, in our population, a large number of horses (164/203, 80.8%) had a BCS of 3 or above and could be classified as normal, fat, or very fat [17]. In horses with a low BCS, a very small proportion of the total, few owners reported or were concerned about weight loss, especially when animals were retired or old: most of them could not determine when the horse started losing weight and considered normal the loss of weight associated with either the equid growing old or with a reduced amount of sweet feed given when horses were no longer in active training. Some of them also correlated the decrease in BCS to the loss of muscle mass secondary to the resting period granted at the end of the sports season. It is worth considering, though, that the low number of horses with a history of weight loss did not allow for a statistical evaluation of the relationship between this condition and gastric ulcers in our population.

The study has some limitations. Firstly, no follow-up information on the improvement or weight gain after treatment for EGUS was available. At the same time, most of the owners of horses with low BCS but free of gastric ulcers declined further tests to determine the cause of the condition (i.e., oral glucose absorption test, rectal biopsy, ultrasound evaluation), considering them either too costly or too invasive, so it was not possible to exclude the presence of other occult diseases. It needs to be stated that animals were presented for evaluation at the request of the owner or referring veterinarian, that could have selected horses considered more at risk or with higher value (both emotional or economical). This selection bias, though, can also be found in a normal clinical setting, where owners and referring vets would be more inclined to present gastroscopy horses with clinical signs or with high value. Furthermore, gastroscopy would not be the first choice, but normally other diagnostic or treatment plans would be implemented before endoscopy, as was the case in the study population. Based on the high number of horses with normal or above normal BCS, though, low BCS was not a selection criterion in this population.

## 5. Conclusions

In our study population of non-athlete horses, a low BCS score was not associated with a greater incidence of gastric ulcers when compared to normal or overconditioned horses. It, therefore, cannot be considered indicative of ESGD or EGGD or ESGD severity, nor should it be used to rule out EGUS. Further studies would be useful in the assessment of a relation between BCS score and gastric lesions and between weight loss and gastric ulcers in different categories of horses.

## Figures and Tables

**Table 1:** Presence of ESGD in the different grades of BCS. ESGD was recorded according to [1,2] and BCS according to [17]. Horses were considered positive for ESGD when at least grade 2 was recorded.

	ESGD 0	ESGD 1	ESGD 2	ESGD 3	ESGD 4	Total
BCS 0	0	0	0	0	0	0
BCS 1	0	0	0	0	0	0
BCS 2	5	5	10	5	13	38
BCS 3	21	19	29	17	30	116
BCS 4	10	6	11	6	15	48
BCS 5	0	0	1	0	0	1
Total	36	30	51	28	58	203

**Table 2:** Presence of EGGD in the different grades of BCS. EGGD was considered positive when any irregularity of the mucosa (hyperemia, erosions, or ulcers) was detected [1,2], and BCS was graded according to [17].

	EGGD Negative	EGGD Positive	Total
BCS 0	0	0	0
BCS 1	0	0	0
BCS 2	25	13	38
BCS 3	91	25	116
BCS 4	35	13	48
BCS 5	0	1	1
Total	151	52	203

## Data Availability

The data are available by sending an e-mail to the corresponding author.

## References

[1] Sykes B.W., Hewetson M., Hepburn R.J., Luthersson N., Tamzali Y. (2015). European College of Equine Internal Medicine Consensus Statement—Equine Gastric Ulcer Syndrome in Adult Horses. J. Vet. Intern. Med..

[2] Rendle D., Bowen M., Brazil T., Conwell R., Hallowell G., Hepburn R., Hewetson M., Sykes B. (2018). Recommendations for the Management of Equine Glandular Gastric Disease. UK-Vet. Equine.

[3] le Jeune S.S., Nieto J.E., Dechant J.E., Snyder J.R. (2009). Prevalence of Gastric Ulcers in Thoroughbred Broodmares in Pasture: A Preliminary Report. Vet. J..

[4] Bertone J. Prevalence of Gastric Ulcers in Elite, Heavy Use Western Performance Horses. Proceedings of the 48th Annual AAEP Convention.

[5] Dionne R.M., Vrins A., Le M., Doucet Y., Paré J. (2003). Gastric Ulcers in Standardbred Racehorses: Prevalence, Lesion Description, and Risk Factors. J. Vet. Intern. Med..

[6] Hewetson M., Tallon R. (2021). Equine Squamous Gastric Disease: Prevalence, Impact and Management. Vet. Med..

[7] van den Boom R. (2022). Equine Gastric Ulcer Syndrome in Adult Horses. Vet. J..

[8] Cate R.E., Nielsen B.D., Spooner H.S., O’Connor-Robison C.I., Schott H.C. (2012). Prevalence of Gastric Ulcers and Relationship to Other Parameters in Standardbred Racehorses. Comp. Exerc. Physiol..

[9] Luthersson N., Nielsen K.H., Harris P., Parkin T.D.H. (2009). The Prevalence and Anatomical Distribution of Equine Gastric Ulceration Syndrome (EGUS) in 201 Horses in Denmark. Equine Vet. J..

[10] Sandin A., Skidell J., Häggström J., Nilsson G. (2000). Postmortem Findings of Gastric Ulcers in Swedish Horses Older than Age One Year: A Retrospective Study of 3715 Horses (1924–1996). Equine Vet. J..

[11] Sgorbini M., Bonelli F., Papini R., Busechian S., Briganti A., Laus F., Faillace V., Zappulla F., Rizk A., Rueca F. (2017). Equine Gastric Ulcer Syndrome in Adult Donkeys: Investigation on Prevalence, Anatomical Distribution, and Severity. Equine Vet. Educ..

[12] Busechian S., Sgorbini M., Orvieto S., Pisello L., Zappulla F., Briganti A., Nocera I., Conte G., Rueca F. (2021). Evaluation of a Questionnaire to Detect the Risk of Developing ESGD or EGGD in Horses. Prev. Vet. Med..

[13] Sanchez L.C. (2018). Disorders of the Gastrointestinal System. Equine Internal Medicine.

[14] Millares-Ramirez E.M., Le Jeune S.S. (2019). Girthiness: Retrospective Study of 37 Horses (2004–2016). J. Equine Vet. Sci..

[15] Niedźwiedź A., Kubiak K., Nicpoń J. (2013). Endoscopic Findings of the Stomach in Pleasure Horses in Poland. Acta Vet. Scand..

[16] Hesta M., Shepherd M. (2021). How to Perform a Nutritional Assessment in a First-Line/General Practice. Vet. Clin. N. Am. Equine Pract..

[17] Carroll C.L., Huntington P.J. (1988). Body Condition Scoring and Weight Estimation of Horses. Equine Vet. J..

[18] Henneke D.R., Potter G.D., Kreider J.L., Yeates B.F. (1983). Relationship between Condition Score, Physical Measurements and Body Fat Percentage in Mares. Equine Vet. J..

[19] Hines M.T. (2018). Clinical Approach to Commonly Encountered Problems. Equine Intern. Med. Fourth Ed..

[20] Baker L.A., Burrows A.M., Nonella K.J., Pipkin J.L., Holmes L.D., McEvers T.J., Tennant T.C., Tisdale Z.M., Voyles A.H., Lawrence T.E. (2020). Relationship between Live Body Condition Score and Carcass Fat Measures in Equine. Transl. Anim. Sci..

[21] Sykes B.W., Bowen M., Habershon-Butcher J.L., Green M., Hallowell G.D. (2019). Management Factors and Clinical Implications of Glandular and Squamous Gastric Disease in Horses. J. Vet. Intern. Med..

[22] Mönki J., Hewetson M., Virtala A.M.K. (2016). Risk Factors for Equine Gastric Glandular Disease: A Case-Control Study in a Finnish Referral Hospital Population. J. Vet. Intern. Med..

[23] Vaccination Guidelines|AAEP. https://aaep.org/guidelines/vaccination-guidelines.

[24] Sykes B.W., Jokisalo J.M. (2014). Rethinking Equine Gastric Ulcer Syndrome: Part 1—Terminology, Clinical Signs and Diagnosis. Equine Vet. Educ..

[25] Begg L.M., O’Sullivan C.B. (2003). The Prevalence and Distribution of Gastric Ulceration in 345 Racehorses. Aust. Vet. J..

[26] Bezděková B., Jahn P., Vyskočil M., Plachý J. (2005). Gastric Ulceration and Exercise Intensity in Standardbred Racehorses in Czech Republic. Acta Vet. Brno.

[27] Jaqueth A.L., Iwaniuk M.E., Burk A.O. (2018). Characterization of the Prevalence and Management of Over-Conditioned Ponies and Horses in Maryland. J. Equine Vet. Sci..

[28] Jensen R.B., Danielsen S.H., Tauson A.H. (2016). Body Condition Score, Morphometric Measurements and Estimation of Body Weight in Mature Icelandic Horses in Denmark. Acta Vet. Scand..

[29] Potter S.J., Bamford N.J., Harris P.A., Bailey S.R. (2016). Prevalence of Obesity and Owners’ Perceptions of Body Condition in Pleasure Horses and Ponies in South-Eastern Australia. Aust. Vet. J..

[30] Stephenson H.M., Green M.J., Freeman S.L. (2011). Prevalence of Obesity in a Population of Horses in the UK. Vet. Rec..

